# Beyond Compliance: The Role of Self-Efficacy in Foot Care and Self-Management Among Patients With Type 1 Diabetes

**DOI:** 10.1155/tswj/8848211

**Published:** 2025-03-18

**Authors:** Mohamad Amir Sadeghi, Afsaneh Raiesifar, Sanaz Aazami

**Affiliations:** Department of Nursing, Faculty of Nursing and Midwifery, Ilam University of Medical Sciences, Ilam, Iran

**Keywords:** chronic conditions, compliance, mediation analysis, self-efficacy, self-management

## Abstract

**Background:** Effective self-management behaviors are crucial for diabetes management. This study examines the mediating role of self-efficacy in the relationship between compliance dimensions and self-management activities in patients with Type 1 diabetes.

**Methods:** The current study explores a baseline analysis from a randomized controlled trial but the intervention's effectiveness is not reported here; the analysis focuses on elucidating potential mediating factors at baseline. The study investigated the relationships between seven compliance dimensions (treatment effort, intention, adaptability, integration, adherence, commitment, and indecisiveness) and six self-management activities (diet, exercise, smoking cessation, blood sugar monitoring, and foot care) through the potential mechanism of self-efficacy.

**Results:** The analysis revealed an indirect association between three compliance dimensions (treatment effort, intention, and commitment) and foot care behavior, mediated by self-efficacy. Additionally, self-efficacy was identified as an indirect mechanism influencing the association between commitment and adaptability with dietary behaviors.

**Conclusions:** This study highlights the importance of self-efficacy in promoting self-management behaviors in chronic conditions. By targeting specific compliance dimensions that influence self-efficacy, healthcare professionals can potentially improve patient self-management.

**Trial Registration:** Iranian Registry of Clinical Trials number: IRCT20221029056335N1

## 1. Introduction

Strict adherence to self-management regimens remains an obstacle in optimizing treatment outcomes for Type 1 diabetes. This is true even among highly motivated patients, highlighting the inherent difficulties associated with the constant needs for therapeutically demanding medications [1]. Type 1 diabetes management strives for a patient-centered equilibrium. This involves effective execution of daily self-care activities to optimize glycemic control while minimizing disease burden on the patient's everyday life [2]. This comprehensive strategy encompasses blood glucose monitoring, medication compliance, multiple insulin injection, the need for carbohydrate counting, participation in regular exercise routines, and the integration of self-care behaviors conducive to overall health [3].

Conceptualized as a preventative approach, self-care encompasses practical, day-to-day activities that contribute to a robust foundation of overall well-being. By prioritizing these behaviors, individuals can proactively mitigate the risk of illness and its enduring consequences [4]. Empirical evidence suggests a positive correlation between proactive self-care behaviors and improved health outcomes [4]. Therefore, for people with diabetes, prioritizing self-care strategies is essential. This helps to minimize the risk of complications and maintain a high quality of life [2, 5]. A paradox exists in Type 1 diabetes management: The acknowledged significance of self-care practices is often overshadowed by low patient compliance [6]. Compliance is a complex and multifaceted construct influenced by a multitude of interacting factors. While self-care practices demonstrably contribute to compliance, they are not solely sufficient to ensure full patient engagement. Internalization of therapeutic recommendations does not necessarily translate to action but ultimately is a patient-driven choice [7]. In this domain of research, self-efficacy emerges as a pivotal construct, significantly predicting compliance to self-care regimens among patients with Type 1 diabetes [8]. Empirical evidence supports a positive association between self-efficacy and successful diabetes management [9, 10]. However, there still is a paradox in the literature: Patients with diabetes often report high self-efficacy for proper foot care, yet exhibit low compliance to preventive behaviors. This discrepancy suggests the need for more analyses of psychological factors influencing actual behaviors to identify conditions that promote increased engagement in preventive foot care [11]. Extant research regarding self-care behaviors and self-efficacy in Type 2 diabetes patients yields inconclusive findings. While some studies report a positive correlation, suggesting higher self-efficacy translates to improved self-care behaviors [12–14], others reveal a weaker or even absent association [15–17]. Nevertheless, pertaining to Type 1 diabetes patients, it has been shown that self-efficacy exerts the strongest influence on compliance to self-care behaviors [18]. To our knowledge, there is no previous study that explains the relationship between self-care practices, self-efficacy, and medication compliance among Type 1 diabetes patients. Therefore, in this study, we aimed to assess the potential mediating effect of self-efficacy on the relationship between different dimensions of compliance to treatment and self-management activities among Type 1 diabetes patients.

## 2. Materials and Methods

### 2.1. Research Setting

The current study explores baseline data from a randomized controlled trial conducted at two teaching hospitals affiliated with Ilam University of Medical Sciences. This study is aimed at assessing the impact of an educational intervention on self-management behaviors in patients with Type 1 diabetes. The trial was registered in the Iranian Registry of Clinical Trials. The current investigation was conducted at two teaching hospitals affiliated with Ilam University of Medical Sciences in Ilam, Iran. These hospitals served as the sole designated centers for providing both outpatient and inpatient diabetes care for Type 1 patients within the region.

### 2.2. Research Design and Participants

The current study explores a baseline analysis from a randomized controlled trial involving patients with Type 1 diabetes. While the intervention's effectiveness is not reported here, the analysis focuses on elucidating potential mediating factors at baseline.

Inclusion criteria for this study were as follows:

• Age between 18 and 65 years old.
• Minimum literacy level sufficient for independent reading and writing.
• Confirmed diagnosis of Type 1 diabetes by a specialist physician at least 6 months prior.
• Absence of chronic psychological or physical illnesses.
• Possession of a mobile phone and access to online messaging applications were required for intervention delivery, as participants received educational materials through these platforms. While this criterion facilitated effective communication, we acknowledge that it may have unintentionally excluded individuals with lower socioeconomic status, which is addressed in the study limitations

Patients were excluded from the study upon self-reported intention to withdraw from the research at any point, if they experienced hospital readmission during the study period, if they encountered significant difficulties performing daily living activities, and if they were unresponsive after three consecutive phone calls from the researcher spaced 1 day apart.

### 2.3. Instrument

#### 2.3.1. Summary of Diabetes Self-Care Activities (SDSCA) Measure

The SDSCA measure is a brief self-report questionnaire of diabetes self-management [19]. This 15-item tool is designed to assess the following aspects of the diabetes regimen: general diet (5 items), specific diet (1 item for insulin injection or pills), exercise (2 items), blood glucose testing (2 items), foot care (4 items), and smoking (1 item). Scoring utilized a 0–7 scale for each behavior, with smoking measured on a 0–1 scale. This yielded a total possible score ranging from 0 to 99. Nevertheless, given the low interitem correlations for this scale, using the individual items is recommended [19]. Therefore, the following instruction was used to score the six dimensions of SDSCA measure:

• General diet = mean number of days for Items 1 and 2.
• Specific diet = mean number of days for Items 3, and 4, reversing Item 4 (0 = 7, 1 = 6, 2 = 5, 3 = 4, 4 = 3, 5 = 2, 6 = 1, and 7 = 0).
• Exercise = mean number of days for Items 5 and 6.
• Blood glucose testing = mean number of days for Items 7 and 8.
• Foot care = mean number of days for Items 9 and 10.
• Smoking status = Item 11 (0 = *nonsmoker*, 1 = *smoker*) and number of cigarettes smoked per day.

Internal consistency of the questionnaire was established using Cronbach's alpha, resulting in a reliability coefficient of 0.78. The instrument was administered via phone call at three time points: preintervention, postintervention at week 6, and follow-up at Week 12.

#### 2.3.2. Self-Efficacy

Self-efficacy was assessed using the Sherer General Self-Efficacy Scale (SGSE) [20]. This 17-item instrument employs a Likert-type response format, ranging from “*completely disagree*” (scored 1) to “*completely agree*” (scored 5). Scores on the SGSE range from a minimum of 17 to a maximum of 85. Total scores were obtained by summing responses across all items. Higher scores reflect a stronger personal belief in one's ability to successfully manage challenges. The SGSE was chosen over the Diabetes Management Self-Efficacy Scale due to its broad applicability in assessing general self-efficacy across chronic conditions. Moreover, the SGSE has been validated for use in Persian-speaking populations, ensuring its reliability in our study setting.

#### 2.3.3. Compliance

The Persian version of *adherence questionnaire* was developed by Fatemi et al. [21] to measure treatment compliance in patients with chronic disease. This 48-item questionnaire was developed in Persian language to measure seven subdimensions including making effort for treatment, intention to take the treatment, adaptability, integrating illness into life, stick to the treatment, commitment to treatment, and indecisiveness for applying treatment. Answers to each item range from “*completely disagree*” (scored 1) to “*completely agree*” (scored 5). The higher score indicates higher compliance with the respective subdimensions.

### 2.4. Statistical Analysis

Statistical analyses were conducted using IBM SPSS Statistics software (version 25). A two-tailed alpha level of 0.05 was employed to determine statistical significance. Descriptive statistics were utilized to summarize the data. The analysis process consisted of two main stages. The analysis of this study commenced with a descriptive presentation of the sample characteristics. This included reporting the mean (average) and standard deviation (a measure of variability) for self-care activities, self-efficacy scores, and medication compliance. The relationships between the study variables were assessed using bivariate correlation analysis.

To evaluate the indirect influence of self-care activities on medication compliance mediated by self-efficacy, this study employed Baron and Kenny's [22] framework for testing mediation pathways. This framework entails three steps. First, it requires a significant association between the seven subdimensions of independent variable (making effort, intention, adaptability, integrating illness into life, compliance, commitment, and indecisiveness for applying treatment) and the dependent variable (seven dimensions of self-care activities). Second, the independent variable must significantly predict the mediator (self-efficacy). Finally, in a model controlling for the independent variable, the mediator must still significantly predict the dependent variable. If all three conditions are met, partial mediation is established. Full mediation is achieved when the independent variable's beta coefficient becomes nonsignificant in the final model containing all three variables. For correlation and mediation analyses using hierarchical linear regression, we reported confidence intervals (95% CIs) and effect sizes (Cohen's *f*^2^ for regression models). Bootstrapped CIs were used where applicable.

### 2.5. Ethical Considerations

Ethical approval for this study was obtained from the ethical committee of the affiliated University of Medical Sciences (Code IR.MEDILAM.REC.1401.134). Additionally, the study was registered with the Iranian Registry of Clinical Trials.

## 3. Results

The study sample comprised 81 participants diagnosed with Type 1 diabetes. The majority of participants were male (54.9%) and married (64.6%). Additionally, a noteworthy portion (25.6%) had attained a bachelor's degree or higher.

We conducted a one-way ANOVA to compare the means of self-management activities between different educational levels. A one-way ANOVA revealed a significant effect of the educational level on blood glucose testing, *F*(5, 75) = 4.359, *p* < 0.001. Tukey's HSD post hoc test showed that mean blood glucose testing of the high school education group was significantly lower than that of the diploma group (*p* = 0.009), associated bachelor group (*p* = 0.001), undergraduate group (*p* = 0.008), and postgraduate group (*p* = 0.001). However, there were no significant differences between the mean blood glucose testing of the other groups.

This study adopted Baron and Kenny's approach [22] to examine the mediating role of self-efficacy in the relationship between medication compliance and self-care activities. Before testing the mediated model, two key assumptions were evaluated:

a. Is there a significant association between seven dimensions of medication compliance and six self-care activities?
b. Is there a significant association between medication compliance and self-efficacy?

Table 1 shows series of significant correlations that establish the first prerequisite:

1. Making effort (*r* = 0.24, *p* < 0.05) was significantly correlated with foot care.
2. Intention to take the treatment (*r* = 0.23, *p* < 0.05) was significantly correlated with foot care.
3. Adoptability (*r* = 0.24, *p* < 0.05) was significantly correlated with specific diet.
4. Commitment to treatment (*r* = 0.42, *p* < 0.001) was significantly correlated with foot care.
5. Commitment to treatment (*r* = 0.42, *p* < 0.001) was significantly correlated with general diet.
6. Commitment to treatment (*r* = 0.37, *p* < 0.001) was significantly correlated with blood glucose monitoring.

Pertaining to the second prerequisite, a series of bivariate correlation was conducted to identify the relationship between self-efficacy and the seven dimensions of compliance. Findings indicated a series of significant correlation between self-efficacy and making effort (*r* = 0.25, *p* = 0.02), intention to take treatment (*r* = 0.26, *p* = 0.02), adoptability (*r* = 0.36, *p* = 0.001), and commitment (*r* = 0.30, *p* = 0.007). Nevertheless, no significant association was found between self-efficacy and integration into daily life, compliance to treatment, and smoking as well indecisiveness.

Therefore, our study is capable of examining *six mediational pathways* (see Numbers 1–6 above) whether self-efficacy mediates the association between four dimensions of the compliance and four of the self-care activities. In order to examine this mediational analysis, a series of hierarchical linear regression was run (Table 2).

In order to examine mediational model, we run six hierarchical linear regression models (Table 2). A regression analysis was conducted to examine the influence of making effort for treatment on foot care, with self-efficacy as a potential mediator. The model employed a hierarchical approach with three steps. Step 1 included control variables such as age, gender, marital status, and education. Step 2 introduced making effort for treatment (antecedent variable), and Step 3 added the mediator (self-efficacy). The analysis revealed a significant mediation effect. Strong self-efficacy (*β* = 0.59, *p* < 0.001) positively impacted foot care. Notably, the effect of making effort for treatment on foot care remained significant even after accounting for control variables (*β* = 0.26, *p* < 0.05 in Step 2). However, the strength of this association (*β*) decreased in Step 3 (*β* = 0.20, *p* < 0.05), suggesting partial mediation. This reduction in the *β* coefficient for making effort for treatment when self-efficacy was included supports the mediating role of self-efficacy in the relationship between making effort for treatment and foot care.

The second pathway is designed to investigate potential influence of self-efficacy on the link between a person's intention to receive treatment and foot care behavior. The findings revealed that self-efficacy had a positive and significant effect on foot care engagement (*β* = 0.59, *p* < 0.001). Interestingly, the intention to receive treatment remained a significant factor influencing foot care even after other variables were accounted for (*β* = 0.25, *p* < 0.05 in Step 2). However, the strength of this association (*β*) slightly decreased when self-efficacy was included in the analysis (*β* = 0.18, *p* < 0.05 in Step 3), suggesting that self-efficacy partially mediates the relationship. This decrease in the influence of intention to receive treatment after incorporating self-efficacy supports the notion that self-efficacy plays a mediating role in the pathway between intention and foot care behavior.

The third pathway tried to assess potential mediating effect of self-efficacy on the linkage between commitment to apply treatment and foot care. Results of the three steps hierarchical regression analysis reported a strong effect of self-efficacy on foot care (*β* = 0.52, *p* < 0.001). A careful consideration into Table 2 shows that within Step 2, commitment to apply treatment after controlling for the effect of background factors significantly (*β* = 0.45, *p* < 0.001) predicts foot care. Furthermore, this association remains significant (*β* = 0.27, *p* < 0.001) at the third step where self-efficacy is also present suggesting a partial mediation.

The fourth pathway investigated the indirect effect of self-efficacy on the relationship between commitment to apply treatment and general diet. As depicted in Table 2, self-efficacy significantly (*β* = 0.31, *p* < 0.01) predicted general diet in the presence of commitment to apply treatment (*β* = 0.38, *p* < 0.001). Thereby, it could be concluded that the association between commitment to apply treatment and general diet is significantly mediated by self-efficacy.

The fifth pathway is designed to investigate potential influence of self-efficacy on the link between a person's commitment to apply treatment and blood glucose monitoring. The findings revealed that self-efficacy had a positive and significant effect on blood glucose monitoring engagement (*β* = 0.86, *p* < 0.001). Interestingly, the commitment to apply treatment remained a significant factor influencing blood glucose monitoring even after other variables were accounted for (*β* = 0.32, *p* < 0.05 in Step 2). However, this association (*β*) appears to be nonsignificant when self-efficacy was included in the analysis (*β* = 0.02, *p* = 0.77 in Step 3), suggesting that self-efficacy fully mediates the relationship. This nonsignificant result in the influence of commitment to apply treatment after incorporating self-efficacy supports the notion that self-efficacy plays a mediating role in the pathway between commitment and blood glucose monitoring.

Finally, regression analysis was conducted to examine the influence of adaptability on specific diet, with self-efficacy as a potential mediator. The model employed a hierarchical approach with three steps. Step 1 included control variables such as age, gender, marital status, and education. Step 2 introduced adaptability (antecedent variable), and Step 3 added the mediator (self-efficacy). The analysis revealed a significant mediation effect. Self-efficacy (*β* = 0.24, *p* < 0.001) positively impacted specific diet. Notably, the effect of adaptability on specific diet remained significant even after accounting for control variables (*β* = 0.25, *p* < 0.05 in Step 2). However, the strength of this association (*β*) decreased in Step 3 (*β* = 0.23, *p* < 0.05), suggesting partial mediation. This reduction in the *β* coefficient for adaptability when self-efficacy was included supports the mediating role of self-efficacy in the relationship between adaptability and foot care. Multicollinearity was assessed using variance inflation factors (VIFs). All VIF values were below 5, indicating no severe multicollinearity among the predictor variables.

In conclusion, out of six tested mediational pathways in this study, five significant partial mediation and one full mediation are established.

## 4. Discussion

The current research investigated whether self-efficacy mediates the influence of various compliance behaviors on self-management activities. Specifically, the study explored how seven dimensions of compliance (e.g., treatment effort, intention, adaptability, integration, compliance, commitment, and indecisiveness) relate to six self-management activities (diet, exercise, smoking cessation, blood sugar monitoring, and foot care) through the potential mechanism of self-efficacy.

In the present study, we used adherence questionnaire developed by Fatemi et al. [21] to measure treatment compliance with chronic disease. This questionnaire is a unique on its own category because of measuring seven important aspects in compliance with the treatment. Compliance is complex phenomenon influenced by a multifaceted interplay of factors exhibiting a reciprocal relationship that can exert significant effect on patients' adherence behaviors. Therefore, elaborating different dimensions of compliance, help us identify its detailed associations with consequent self-care activities.

The results of this study revealed an indirect association between three compliance dimensions (making effort, intention, and commitment) and foot care behavior, mediated by self-efficacy. This finding may justify previous evidences on the lack of association between foot care self-efficacy belief with actual foot care behaviors [11, 23]. The current findings highlight the importance of perceived self-efficacy in making effort to adhere into self-foot care in performing actual foot care practices. Elucidating factors that hinder optimal foot care behaviors is crucial for achieving significant reductions in subsequent outcome [11]. Individuals with higher self-efficacy exhibit a stronger perception of self-management capability, leading them to invest greater effort in self-care behaviors. Conversely, lower self-efficacy likely translated into a diminished sense of capability, potentially leading to early abandonment of self-management practices [24]. This study identified intention and commitments as the key subdimensions of compliance that influence self-care management behaviors among patients with Type 1 diabetes. Similarly, strong intentions were found to promote preventive actions related to diabetic ulcers. This suggests that a patient's belief in the benefits of preventing ulcers translates into a conscious effort to avoid them [25]. On the other hand, this study adds to the knowledge by finding the indirect association originating from commitment to medication as an antecedent affecting foot care behaviors via self-efficacy. Accordingly, patients' higher appreciation of the role of adherence was shown to result in higher commitment to using therapeutic footwear after healing and consequently to reduced relocation rates [26].

Notably, our study identified self-efficacy as a full mediator in the relationship between commitment and blood glucose monitoring. Consistently, a growing body of research suggests that interventions drawing on the principles of commitment theory can enhance patient adherence to medical regimens. This framework holds promise for guiding future investigations into adherence behavior [27]. A possible explanation could be the fact that self-monitoring of blood glucose is primarily a behavioral intervention. Investigations have revealed a link between lower self-monitored blood glucose (SMBG) frequency and specific psychological factors. Individuals with lower self-esteem, self-efficacy, and perceived competence in managing their diabetes are more likely to exhibit reduced SMBG frequency [28]. For individuals with high HbA1c levels, identifying modifiable behavioral factors could be a valuable tool in optimizing HbA1c management strategies. This approach has the potential to enhance treatment effectiveness, thereby reducing the incidence of both acute and chronic diabetic complications [29]. Hence, this study adds to the literature by highlighting the importance of commitment as a behavioral aspect in diabetes management.

Another key finding of this study was the identification of self-efficacy as an indirect mechanism influencing the relationship between two compliance dimensions (commitment and adaptability) and dietary behaviors. This finding represents a novel contribution to the existing knowledge, as it is the first to identify this specific indirect effect, particularly within the context of Type 1 diabetes management. Individuals with higher self-efficacy in self-management exhibited improved compliance to diet and medication regimens and a greater propensity to engage in regular exercise programs [24]. Within the context of behavior modification, healthcare providers play a crucial role in equipping patients with the skills necessary to manage their condition, particularly during periods of disrupted routines due to treatment limitations. In this context, adaptability, a core component of patient empowerment, facilitates the adoption of new routines and fosters help-seeking behavior [30]. Prior research on Type 2 diabetes patients has consistently shown a negative correlation between self-efficacy and compliance to various self-management behaviors, including medication use, dietary practices, exercise routines, blood glucose monitoring, and foot care [31, 32].

Our findings revealed significant differences in blood glucose testing behaviors based on educational level, with individuals possessing higher educational attainment demonstrating greater self-efficacy in diabetes self-management. This highlights the need for tailored diabetes education programs that address the unique needs of individuals with lower educational levels. Specifically, healthcare providers could design simplified, accessible, and culturally sensitive educational materials to enhance self-efficacy among less-educated patients. Additionally, integrating visual aids, hands-on demonstrations, and peer support groups may help bridge the gap in self-management behaviors. These results suggest that policymakers should prioritize diabetes education as part of national healthcare strategies, particularly targeting populations with lower education levels. Efforts could include subsidizing community-based diabetes education programs or making digital health platforms more accessible. Ensuring equity in healthcare education resources may mitigate disparities in self-management outcomes among patients with varying educational backgrounds.

### 4.1. Limitation

The main research limitation in this cross-sectional analysis is the inability to establish causal direction between the variables. The present study acknowledges the inherent limitations associated with self-reported data, which can introduce bias and potentially compromise the accuracy of inferences drawn about variable relationships. To mitigate this concern, we employed well-constructed questionnaires specifically designed to minimize social desirability bias and enhance recall accuracy. We recognize that religious piety could play a role in shaping patients' attitudes toward self-management. For example, some individuals may attribute their health outcomes to divine intervention, potentially affecting their adherence to self-care practices. Future research should explore the impact of religious beliefs on self-efficacy and compliance in chronic disease management, particularly in culturally diverse populations. In addition to self-efficacy, factors such as psychological distress, social support, and financial constraints may influence diabetes self-management. Future research should consider these variables to gain a more comprehensive understanding of patient adherence and engagement. Another limitation of this study is the requirement for participants to possess a mobile phone and have access to online messaging applications. While this facilitated effective communication and ensured timely intervention delivery, it may have inadvertently excluded individuals from lower socioeconomic backgrounds who lack access to such resources. Future research should explore alternative methods of intervention delivery to ensure inclusivity across diverse socioeconomic groups.

## 5. Conclusion

This study examined the mediating role of self-efficacy in the relationship between various compliance subdimensions and self-management behaviors in patients with chronic conditions. Specifically, it investigated how seven dimensions of compliance, including treatment effort, intention, adaptability, integration, overall compliance, commitment, and indecisiveness, influence six self-management activities (diet, exercise, smoking cessation, blood sugar monitoring, and foot care) through the potential mechanism of self-efficacy. This study identified an indirect effect of three compliance dimensions—treatment effort, intention, and commitment—on foot care behaviors, mediated by self-efficacy. Furthermore, the study revealed self-efficacy as an indirect mechanism underlying the association between two compliance dimensions, commitment and adaptability, and dietary behaviors. Notably, our study identified self-efficacy as a full mediator in the relationship between commitment and blood glucose monitoring.

## Figures and Tables

**Table 1:** Descriptive statistics and inter correlation of variables.

	**Glucose monitoring**	**Foot care**	**Physical activity**	**Specific diet**	**General diet**	**Special diet**	**Exercise**	**Self-efficacy**	**Education**
Making effort	−0.075	−0.24⁣^∗^	0.03	0.19	0.18	0.06	0.03	0.25⁣^∗^	
Intention	−0.123	−0.23⁣^∗^	0.05	0.16	0.20	0.01	0.05	0.27⁣^∗^	
Adoptability	−0.059	−0.13	−0.05	0.24⁣^∗^	0.16	0.09	−0.05	0.36⁣^∗∗^	
Integration	−0.063	−0.17	0.01	0.03	0.11	0.02	0.10	0.06	
Compliance	0.063	−0.21	0.13	0.20	0.10	−0.02	0.13	0.01	
Commitment	−0.117	−0.423⁣^∗∗^	0.189	0.031	0.289⁣^∗∗^	0.053	0.189	−0.299⁣^∗∗^	
Indecisiveness	0.290⁣^∗∗^	−0.097	0.034	0.138	−0.126	−0.09	0.034	0.01	

⁣^∗^Significant at *p* < 0.05.

⁣^∗∗^Significant at *p* < 0.001.

**Table 2:** Mediational analysis for the indirect effect of self-efficacy on the relationship between subdimensions of compliance on self-care activities.

			**Step 1**				**Step 2**	**Step 3**	
Making effort < self-efficacy < foot care			Sex	Age	Education	Marital status	Making effort	Making effort	Self-efficacy
	Beta		0.06	−0.06	0.03	0.10	−0.26	−0.20	0.59
	t		0.50	−0.37	0.29	0.57	−2.25	−2.14	6.10
	Sig.		0.62	0.71	0.78	0.57	**0.03**	**0.04**	**0.00**
	95% CI	Lower	−0.80	−0.75	−0.33	−1.12	−0.20	−0.16	0.16
		Upper	1.33	0.52	0.44	2.02	−0.01	−0.01	0.32

Intention to take treatment < self-efficacy < foot care	t		0.06	−0.06	0.03	0.10	−0.25	−0.18	0.59
	Sig.		0.50	−0.37	0.29	0.57	−2.12	−1.89	6.05
	95% CI	Lower	0.62	0.71	0.78	0.57	**0.04**	**0.05**	**0.00**
		Upper	−0.80	−0.75	−0.33	−1.12	−0.22	−0.17	0.16
			1.33	0.52	0.44	2.02	−0.01	0.00	0.32

Commitment < self-efficacy < foot care			Sex	Age	Education	Marital status	Commitment	Commitment	Self-efficacy
	Beta		0.06	−0.06	0.03	0.10	−0.45	−0.27	0.52
	t		0.50	−0.37	0.29	0.57	−4.13	−2.68	5.10
	Sig.		0.62	0.71	0.78	0.57	**0.00**	**0.01**	**0.00**
	95% CI	Lower	−0.80	−0.75	−0.33	−1.12	−0.40	−0.28	0.13
		Upper	1.33	0.52	0.44	2.02	−0.14	−0.04	0.29

Commitment < self-efficacy < general diet			Sex	Age	Education	Marital status	Commitment	Commitment	Self-efficacy
	Beta		0.04	0.29	0.16	−0.39	0.27	0.38	0.31
	t		0.38	1.80	1.43	−2.36	2.37	3.26	2.71
	Sig.		0.71	0.08	0.16	0.02	**0.02**	**0.00**	**0.01**
	95% CI	Lower	−0.35	−0.03	−0.04	−1.38	0.01	0.04	0.01
		Upper	0.51	0.49	0.27	−0.12	0.12	0.15	0.09

Commitment < self-efficacy < blood glucose monitoring			Sex	Age	Education	Marital status	Blood glucose monitoring	Blood glucose monitoring	Self-efficacy
	Beta		0.04	−0.21	0.31	0.19	−0.32	−0.02	0.86
	t		0.36	−1.37	2.80	1.19	−2.96	−0.29	15.15
	Sig.		0.72	0.18	0.01	0.24	**0.00**	0.77	**0.00**
	95% CI	Lower	−1.38	−1.70	0.25	−1.01	−0.54	−0.13	0.52
		Upper	2.00	0.32	1.48	4.00	−0.11	0.10	0.68

Adoptability > self-efficacy < specific diet			Sex	Age	Education	Marital status	Specific diet	Specific diet	Self-efficacy
	Beta		0.04	0.03	0.18	−0.09	0.25	0.23	0.45
	t		0.31	0.20	1.50	−0.51	2.14	2.33	4.27
	Sig.		0.76	0.84	0.14	0.62	**0.04**	**0.02**	**0.00**
	95% CI	Lower	−0.73	−0.46	−0.08	−1.60	0.01	0.01	0.08
		Upper	1.00	0.57	0.55	0.95	0.19	0.17	0.22

*Note:* Values in boldface indicate significant level < 0.05.

## Data Availability

The data that support the findings of this study are available from the corresponding author upon reasonable request.
